# Changes in second-line regimen durability and continuity of care in relation to national ART guideline changes in South Africa

**DOI:** 10.7448/IAS.19.1.20675

**Published:** 2016-12-23

**Authors:** Dorina Onoya, Alana T Brennan, Rebecca Berhanu, Liudmyla van der Berg, Thulasizwe Buthelezi, Matthew P Fox

**Affiliations:** ^a^ Health Economics and Epidemiology Research Office, Department of Internal Medicine, School of Clinical Medicine, Faculty of Health Sciences, University of the Witwatersrand, Johannesburg, South Africa; ^b^ Department of Global Health, Boston University School of Public Health, Boston, MA, USA; ^c^ Department of Epidemiology, Boston University School of Public Health, Boston, MA, USA; ^d^ Right to Care, Johannesburg, South Africa

**Keywords:** HIV treatment, second-line regimen, South Africa, antiretroviral therapy, drug substitution, treatment durability, treatment interruption.

## Abstract

**Introduction**: Little is known about the impact of antiretroviral therapy (ART) guideline changes on the durability of second-line ART and continuity of care. This study examines predictors of early drug substitutions and treatment interruptions using a cohort analysis of HIV positive adults switched to second-line ART between January 2004 and September 2013 in Johannesburg, South Africa.

**Methods**: The main outcomes were having a drug substitution or treatment interruption in the first 24 months on second-line ART. Kaplan Meiers analyses and Cox proportional hazards regression were used to identify predictors of drug substitutions and treatment interruptions.

**Results**: Of 3028 patients on second-line ART, 353 (11.7%) had a drug substitution (8.6 per 100PY, 95% CI: 7.8–9.6) and 260 (8.6%) had a treatment interruption (6.3 per 100PY, 95% CI: 5.6–7.1). While treatment interruptions decreased from 32.5 per 100PY for the 2004 cohort to 2.3 per 100PY for the 2013 cohort, the rates of drug substitutions steadily increased, peaking at an incidence of 26.7 per 100PY for the 2009 cohort and then decreased to 4.2 per 100PY in the 2011 cohort. Compared to the 2004 to 2008 cohorts, the hazard of early drug substitutions was highest among patients switched to AZT + ddI + LPVr in 2009 to 2010 (aHR 5.1, 95% CI: 3.4–7.1) but remained low over time among patients switched to TDF + 3TC/FTC + LPVr or AZT/ABC + 3TC + LPVr. The main common predictor of both treatment interruption and drug substitution was drug toxicity.

**Conclusions**: Our results show a rapid transition between 2004 and 2010 ART guidelines and concurrent improvements in continuity of care among second-line ART patients. Drug toxicity reporting and monitoring systems need improvements to inform timely regimen changes and ensure that patients remain in care. However, reasons for drug substitutions should be closely monitored to ensure that patients do not run out of treatment options in the future.

## Introduction

With approximately 2.5 (41.6%) of 6 million HIV-infected South Africans currently on antiretroviral therapy (ART) [1] and the number of patients failing first-line ART expected to further increase in the coming years, more and more patients will need the more expensive protease inhibitor (PI)-based second-line regimen [2,3]. Between 17% and 25% of all patients who initiate ART in South Africa will experience virologic (VL) failure within five years [2,4,5]. Data from ART sites in Johannesburg indicate that among patients failing first-line ART, just over 60% are switched to a second-line regimen, equivalent to about 10% of patients started on ART [2,6].

Although new drugs for HIV treatment are being developed, HIV treatment options within national programmes remain limited. Therefore the durability of second-line regimens is critical to the long-term success of ART and ultimately patient survival. In many low and middle income countries (LMIC) with no third-line regimens available, second-line ART is the last option for patients [7]. In South Africa, while third-line ART is defined in national ART guidelines, access is currently limited due to the substantially higher cost [3]. An estimated 33% to 40% of patients are expected to fail treatment in the first 12 months of second-line ART [8–11]. Fortunately VL failure on second-line ART does not necessarily warrant a switch to a third-line regimen as PIs have a high genetic barrier to resistance mutations, particularly among PI naïve patients [10,12,13]. As failure on second-line ART is more related to suboptimal adherence than drug resistance [11,14,15], the management of VL failure on second-line typically involves intensified adherence counselling with drug substitutions recommended in cases of documented resistance mutations, severe adverse drug reactions (ADR) or drug interactions [16–18].

The South African ART guidelines were modified in 2010, 2013 and updated in 2015 to align with global recommendations [19–22]. A major part of these changes was to increase the selection of available second-line regimens and remove didanosine (ddI) from second-line ART [19–22]. While rates of drug substitutions and the durability of first-line ART have been previously described, there is very little information on patterns of drug substitutions for patients on second-line ART across ART guideline-specific periods [23].

Continuity of care is closely associated with positive ART outcomes [24]. Unfortunately patients often interrupt their treatment to deal with drug toxicities, particularly early in the treatment programme when alternatives to more toxic drugs like stavudine (d4T) were limited. Such interruptions increase the risk of viral rebound, development of drug resistance and immunologic failure [24]. In South Africa, 12.8% of patients accessing ART between 2004 and 2009 had a treatment interruption [25]. The introduction of alternatives to d4T and ddI as well as fixed dose antiretroviral combinations in recent years should, in principle, have resulted in a decline in toxicity related treatment interruptions over the years.

We set out to examine patterns and predictors of early drug substitutions and treatment interruptions over the first two years on second-line ART for patients initiated on second-line ART between 2004 and 2013.

## Methods

### Study population

This was an analysis of anonymized electronic medical records of HIV infected adults (≥18 years at ART initiation) switched to standard second-line ART (defined as a triple ART including a PI) after initiating a standard first-line regimen between January 2004 and September 2013 at clinics in Johannesburg, South Africa. We included patients from three non-governmental organization (NGO)-run clinics, four public community health centres (CHCs) and three HIV clinics embedded in public hospitals that receive HIV care and treatment technical support from Right to Care (RTC), a non-profit organization. CHCs are mainly nurse-run with the support of one medical doctor while hospital-based and NGO-run clinics often have more doctors available. The management of patients in both the public and NGO clinics follows national HIV treatment guidelines [19–22].

**Figure 1.**
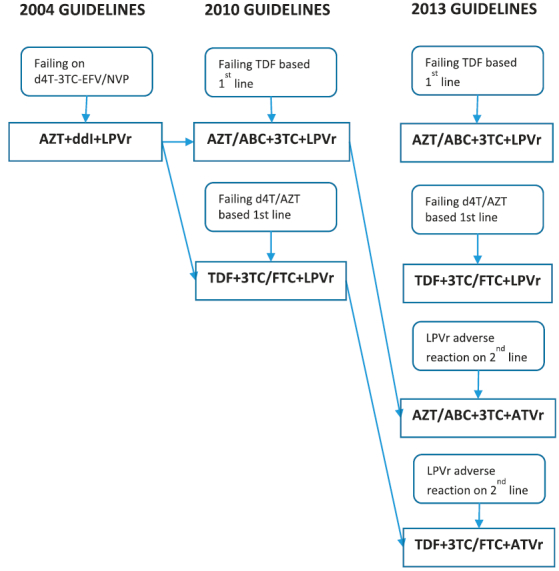
Second-line ART guideline changes in South Africa from 2004 to 2013.

Before April 2010, the primary second-line regimen in South Africa included two nucleotide reverse transcriptase inhibitors (NRTIs) (zidovudine (AZT) and ddI) and ritonavir-boosted lopinavir (LPVr) as the PI component. In 2010, ddI was replaced by new NRTIs (tenofovir (TDF) and abacavir (ABC)) alongside LPVr. In 2013, ritonavir-boosted atazanavir (ATVr) was also added as a PI option along with LPVr [19–21] (Figure 1). Lamivudine (3TC) and emtricitabine (FTC) remain important components of second-line regimen.

Until 2013, HIV positive women with high CD4 counts who became pregnant while stable on first-line ART, substituted efavirenz (EFV) with LPVr as part of first-line ART [22] and then the previous first-line regimen was restored at the end of the pregnancy. We did not consider the initiation of a PI-based regimen during pregnancy as a permanent switch to second-line ART.

Clinical data from the ART clinics were captured by employees of RTC on site and stored on an electronic patient management system, TherapyEdge-HIV^TM^. Additional clinical and laboratory data were obtained from electronic records from the National Health Laboratory Services (NHLS). Data were fully anonymized for analyses. Ethics approval for the retrospective data review was obtained from the Human Research Ethics Committee of the University of Witwatersrand (M140201) as well as Boston University Institutional Review Board (H-29768). In accordance with Section 3 of the recommendations regarding the provisions for waiver or alteration of the informed consent requirements under the South African Department of Health and Human Services (HHS) Regulations at 45 CFR 46.116(d) and the Declaration of Helsinki, a waiver for individual patient consent was obtained.

### Analytic variables

The primary outcome was a drug substitution in the first two years (early) after initiating second-line ART that did not constitute a switch to a third-line regimen. Third-line was defined as any regimen including darunavir (DRV), etravirine (ETR) or raltegravir (RAL). We firstly hypothesized that rates of early drug substitutions would be lower among patients initiated on second-line under the 2010 and 2013 ART guidelines compared to the 2004 guidelines as better second-line regimens were made available. Participants were therefore categorized into annual cohorts based on the calendar year of switch to second-line ART (primary exposure). These were grouped into 2004 to 2008, 2009 to 2010 and 2011 to 2013 periods.

Additionally, we hypothesized that changes in ART guidelines would have resulted in decreased treatment interruptions over time as alternative drugs became available. Treatment interruption was measured as gaps between the initial second-line regimen end date and new regimen start date. Participants were considered to have interrupted treatment if this gap was ≥7 days.

We defined baseline as the time of initiating second-line ART. Variables collected at baseline included: (i) demographic variables; (ii) clinical and laboratory variables (e.g. WHO stage, body mass index (BMI), CD4 counts, viral load and haemoglobin level); (iii) treatment variables (e.g. ART regimen, treatment start and stop date); (iv) measures of liver function (aspartate transaminase (AST)/alanine transaminase (ALT) ratio). These indicators were considered baseline if they were taken between 6 months before and 3 months after initiation of second-line ART.

BMI was categorized as underweight (BMI < 18.5), normal (18.5 ≤ BMI < 25), overweight (25 ≤ BMI < 30) and obese (BMI ≥ 30). Anaemia was defined as a haemoglobin (Hb) value below 13.0 g/dl in men and below 11.5 g/dl in women. VL failure during the observation period was defined as having two consecutive viral load measurements >1000 copies/ml, ≥3 months apart and 3 months after the date of second-line regimen initiation. Possible ADR during follow-up were obtained from clinical visit data [16]. ALT and AST were measured in units per litre (U/l). AST/ALT ratio values were categorized as <1, 1 to 1.9 and 2 or higher.

### Follow-up time

Person time accrued from the date of second-line ART initiation until the outcome of interest, completion of the two years on second-line ART, the last date seen at the clinic during the first two year (for those who died, were lost to follow up or transferred out) or 30 August 2015 (administrative censoring). Loss to follow-up was defined as being ≥3 months late for a scheduled visit.

### Statistical analysis

Data analysis was conducted using STATA version 14 (StataCorp, College Station, Texas). Continuous variables were described using medians and interquartile ranges. Categorical variables were described using percentages. Kaplan Meier analyses were conducted for each outcome of interest. Predictors of drug substitutions/treatment interruptions were modelled using Cox Proportional Hazards model. Variables with a *p* value <0.1 in crude analyses were entered in the multivariate model. Schoenfeld residuals were used to test the assumption of proportional hazards. Interaction terms with time varying covariates were created for variables that violated the proportional hazards assumption. Variables were excluded from the model when the inclusion of the interaction term did not resolve the proportional hazards assumption violation, except for the initial second-line regimen, in which case the model was stratified. Missing data was accounted for by including a ‘not measured/missing’ category where necessary.

## Results

### Cohort description and baseline characteristics

Table 1 shows the distribution of baseline demographic and clinical characteristics of the cohort. Of the 84,215 patients who initiated first-line ART before September 2013 at the 10 clinics, 3028 (3.6%) met the eligibility criteria and were included in the analysis. Overall 66.2% of eligible patients were female. At the time of switching to second-line, over 80% were 30 years or older. The majority of patients were unemployed (58.8%). The initial second-line regimens represented in this population were AZT + ddI + LPVr (20.4%), AZT/ABC + 3TC + LPVr (36.4%), TDF + 3TC/FTC + LPVr (42.3%) and recommended ATVr-based regimen (0.9%). Only 10% of patients were at least 3 months late for a scheduled visit during the observation period. Overall, 43.4% of patients had a documented VL failure before switching to second-line ART, and 45.1% had experienced an ADR before the switch. Among those who had a VL failure on first-line ART, 43.0% had also experienced an ADR. However only 6.7% had a VL failure and 13.8% experienced an ADR during follow-up.

**Table 1.** Demographic and clinical characteristics of study cohort by period of second-line ART initiation.

	2004 to 2008	2009 to 2010	2011 to 2013	Total
	*N* = 489	*N* = 726	*N* = 1813	*N* = 3028
	*n* (col %)	*n* (col %)	*n* (col %)	*n* (col %)
**Gender**				
Female	340 (69.5)	489 (67.4)	1177 (64.9)	2006 (66.2)
Male	149 (30.5)	237 (32.6)	636 (35.1)	1022 (33.8)
**Age**				
Under 25	34 (7.0)	40 (5.5)	121 (6.7)	195 (6.4)
25 to 29.9	80 (16.4)	99 (13.6)	211 (11.6)	390 (12.9)
30 to 39.9	237 (48.5)	345 (47.5)	778 (42.9)	1360 (44.9)
40 to 49.9	98 (20.0)	181 (24.9)	510 (28.1)	789 (26.1)
≥50	40 (8.2)	61 (8.4)	193 (10.6)	294 (9.7)
**Clinic type**				
HIV clinic in hospital complex	346 (70.8)	413 (56.9)	821 (45.3)	1580 (52.2)
Local CHC	14 (2.9)	115 (15.8)	328 (18.1)	457 (15.1)
NGO clinic	129 (26.4)	198 (27.3)	664 (36.6)	991 (32.7)
**Initial second-line regimen**				
AZT + ddI + LPVr	334 (68.3)	278 (38.3)	6 (0.3)	618 (20.4)
AZT/ABC + 3TC + LPVr	94 (19.2)	168 (23.1)	839 (46.3)	1101 (36.4)
TDF + 3TC/FTC + LPVr	47 (9.6)	278 (38.3)	956 (52.7)	1281 (42.3)
ATVr regimen	14 (2.9)	2 (0.3)	12 (0.7)	28 (0.9)
**VL failure on first-line ART**				
Yes	176 (36.0)	231 (31.8)	906 (50.0)	1313 (43.4)
No	313 (64.0)	495 (68.2)	907 (50.0)	1715 (56.6)
**Possible ADR on first-line ART**				
Yes	269 (55.0)	361 (49.7)	735 (40.5)	1365 (45.1)
No	220 (45.0)	365 (50.3)	1078 (59.5)	1663 (54.9)
**WHO stage at second-line ART initiation**				
I or II	318 (65.0)	504 (69.4)	960 (53.0)	1782 (58.9)
III or IV	94 (19.2)	138 (19.0)	317 (17.5)	549 (18.1)
Not measured/missing	77 (15.7)	84 (11.6)	536 (29.6)	697 (23.0)
**CD4 at second-line ART initiation**				
199.9 cells/µl or less	230 (47.0)	262 (36.1)	518 (28.6)	1010 (33.4)
200 to 349.9 cells/µl	133 (27.2)	227 (31.3)	361 (19.9)	721 (23.8)
350 cells/µl or higher	96 (19.6)	154 (21.2)	402 (22.2)	652 (21.5)
Not measured	30 (6.1)	83 (11.4)	532 (29.3)	645 (21.3)
**BMI at second-line ART initiation**				
Underweight	37 (7.6)	62 (8.5)	153 (8.4)	252 (8.3)
Normal	213 (43.6)	325 (44.8)	786 (43.4)	1324 (43.7)
Overweight	118 (24.1)	182 (25.1)	424 (23.4)	724 (23.9)
Obese	59 (12.1)	121 (16.7)	294 (16.2)	474 (15.7)
Not measured/missing	62 (12.7)	36 (5.0)	156 (8.6)	254 (8.4)
**Anaemia at second-line ART initiation**				
Yes	91 (18.6)	121 (16.7)	301 (16.6)	513 (16.9)
No	365 (74.6)	520 (71.6)	1037 (57.2)	1922 (63.5)
Not measured/missing	33 (6.7)	85 (11.7)	475 (26.2)	593 (19.6)
**AST to ALT ratio at second-line ART initiation**				
<1	71 (14.5)	120 (16.5)	72 (4.0)	263 (8.7)
1 to 1.9	273 (55.8)	337 (46.4)	189 (10.4)	799 (26.4)
2 or higher	44 (9.0)	60 (8.3)	44 (2.4)	148 (4.9)
Not measured/missing	101 (20.7)	209 (28.8)	1508 (83.2)	1818 (60.0)
**Virologic (VL) failure in follow-up**				
Yes	35 (7.2)	36 (5.0)	133 (7.3)	204 (6.7)
No	454 (92.8)	690 (95)	1680 (92.7)	2824 (93.3)
**≥3 months late for scheduled visit in follow-up**				
Yes	86 (17.6)	90 (12.4)	144 (7.9)	320 (10.6)
No	403 (82.4)	636 (87.6)	1669 (92.1)	2708 (89.4)
**Possible ADR in follow-up**				
Yes	147 (30.1)	104 (14.3)	167 (9.2)	418 (13.8)
No	342 (69.9)	622 (85.7)	1646 (90.8)	2610 (86.2)

### Descriptions of observed drug substitutions

Table 2 describes the drug substitutions in this sample as well as possible ADR by initial second-line regimen. Overall 11.7% (*n* = 353) of patients had an early drug substitution at a median time of 10.6 months (IQR: 4.6–17.5). Overall 30% of patients who started on AZT + ddI + LPVr had a drug substitution and 96.5% (167/173) of substitutions involved swapping out ddI. Only 6.5% of patients initiated on AZT/ABC + 3TC + LPVr had a substitution and 61.1% (44/72) of cases involved swapping out AZT. Among patient initiated on TDF + 3TC/FTC + LPVr for second-line, 8.4% had a substitution and 46.7% (50/107) of cases involved swapping out TDF and 30 (28.0%) involved swapping out LPVr.

**Table 2.** Description of drug substitutions in the first two years of second-line ART by initial second-line ART regimen.

	AZT + ddI + LPVr	AZT/ABC + 3TC + LPVr	TDF + 3TC/FTC + LPVr	ATVr regimen	Total	Time to drug substitution
	*N* = 173	*N* = 72	*N* = 107	*N* = 1	*N* = 353	Median months (IQR)
	*n* (col %)	*n* (col %)	*n* (col %)	*n* (col %)	*n* (col %)	
**Outgoing drug**						
3TC/EMT	0	1 (1.4)	27 (25.2)	0	28 (7.9)	18.8 (10.3–21.8)
ABC	0	3 (4.2)	0	0	3 (0.9)	3.7 (0.5–14.0)
AZT	3 (1.7)	44 (61.1)	0	0	47 (13.3)	4.8 (1.8–14.9)
ddI	167 (96.5)	0	0	0	167 (47.3)	12.9 (7.9–17.9)
LPVr	3 (1.7)	24 (33.3)	30 (28.0)	0	57 (16.2)	10.3 (4.5–16.1)
TDF	0	0	50 (46.7)	1 (100)	51 (14.5)	4.8 (1.9–11.6)
**Possible ADR**						
None reported	121 (69.9)	54 (75.0)	89 (83.2)	1 (100)	265 (75.1)	10.3 (4.6–17.0)
Anaemia	2 (1.2)	4 (5.6)	1 (0.9)	0	7 (2.0)	3.3 (1.1–15.6)
Breast condition	0	0	1 (0.9)	0	1 (0.3)	10.6 (10.6–10.6)
Gastric condition	9 (5.2)	6 (8.3)	4 (3.7)	0	19 (5.4)	12.9 (7.9–18.4)
Kidney problems	1 (0.6)	0	1 (0.9)	0	2 (0.6)	9.0 (1.9–16.0)
Lactic condition	6 (3.5)	1 (1.4)	0	0	7 (2.0)	4.1 (2.1–14.8)
Lipid conditions	15 (8.7)	4 (5.6)	3 (2.8)	0	22 (6.2)	15.5 (11.0–19.8)
Liver condition	4 (2.3)	0	3 (2.8)	0	7 (2.0)	5.8 (5.5–21.5)
Neuropathy	5 (2.9)	1 (1.4)	4 (3.7)	0	10 (2.8)	9.8 (4.3–15.6)
Skin condition	7 (4.1)	2 (2.8)	1 (0.9)	0	10 (2.8)	6.6 (2.1–14.0)
Sleep problems	3 (1.7)	0	0	0	3 (0.9)	8.9 (8.0–21.1)
Time to ADR, median months (IQR)	12.9 (7.8–17.9)	6.3 (2.1–15.0)	9.2 (3.8–18.6)	4.4	10.6 (4.6–17.5)	

Substitutions of ABC, AZT or TDF were made in the first 6 months after switching to second-line ART (Median: 3.7, 4.8 and 4.8 months, respectively), ddI and LPVr substitutions were made over six months after switch (Median 12.9 and 10.3, respectively). Only 88 (24.9%) of patients with drug substitutions had a possible ADR.

### Drug substitutions and treatment interruptions over time

**Figure 2.**
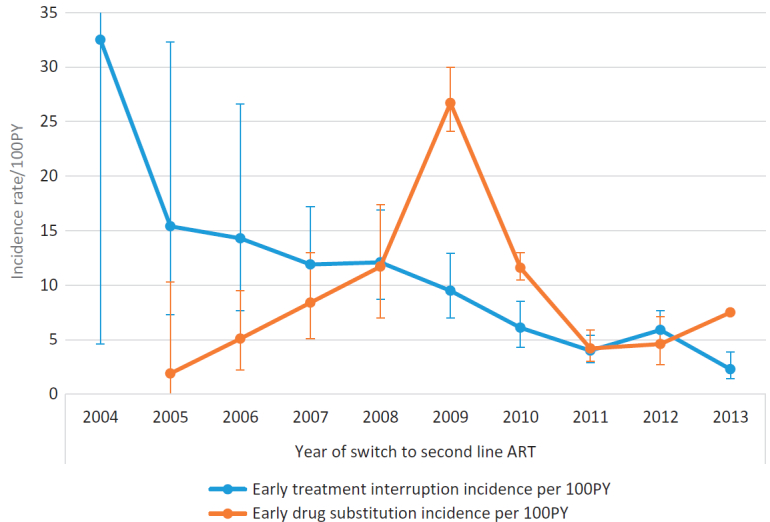
Incidence rate of drug substitutions/treatment interruption in the first two years on second line ART by year of initial second-line ART regimen.

**Figure 3.**
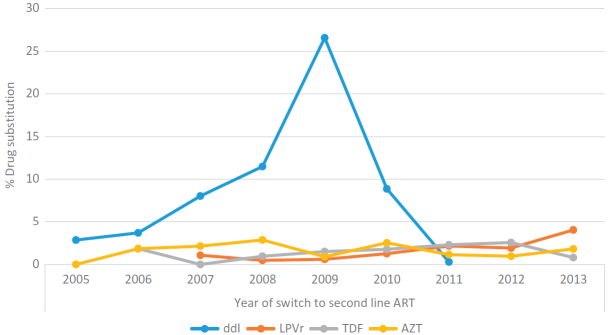
Proportion of annual cohort with drug substitutions in the first two years on second line ART by outgoing ARV drug.

Figure 2 shows the incidence for drug substitutions and treatment interruptions across annual second-line cohorts, and Figure 3 shows the proportion of the annual cohorts with a substitution by the outgoing drug. Overall 353 (11.7%) patients experienced an early drug substitution at a rate of 8.6 per 100 person years (PY) (95% CI: 7.8–9.6). A lower proportion of patients experienced a treatment interruption (260 or 8.6%) at a rate of 6.3 per 100PY (95% CI: 5.6–7.1). While treatment interruptions have decreased gradually from a high incidence rate of 32.5 per 100PY for the 2004 cohort to 2.3 per 100PY for the 2013 cohort, the rates of drug substitutions steadily increased, peaking at an incidence of 26.7 per 100PY for the 2009 cohort and then decreased to 4.2 per 100PY in the 2011 cohort. Similarly the proportion of patients with a ddI substitution gradually increased from 2.9% for the 2005 cohort to 26.6% in the 2009 cohort (Figure 3). Substitutions of AZT, TDF or LPVr remained relatively low over time.

### Predictors of drug substitutions

**Figure 4.**
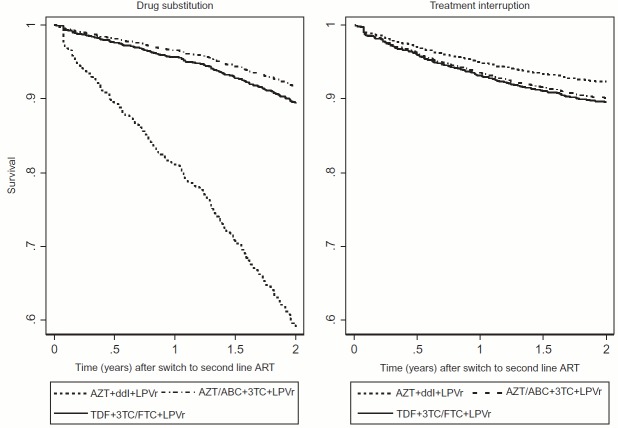
Survival curves for drug substitutions and treatment interruptions in the first two years on second line ART by initial second line ART regimen.

Table 3 presents crude (HR) and adjusted hazards ratios (aHR) of drug substitutions on second-line. The initial second-line regimen was an important predictor of drug substitution (Figure 4). Compared to patients switched to AZT + ddI + LPVr, those initiated second-line ART on AZT/ABC + 3TC + LPVr (aHR 0.2, 95% CI: 0.1–0.4) or TDF + 3TC/FTC + LPVr (aHR 0.2, 95% CI: 0.1–0.8) had a lower risk of drug substitution.

**Table 3.** Demographic and clinical predictors of drug substitutions in the first two years on second-line ART.

	Crude associations				Adjusted associations, aHR (95% CI)			
	Person-time	Failures, *n* (%)	Incidence rate/100PY (95% CI)	HR (95% CI)	Total sample	Stratified by initial second-line regimen		
						AZT + ddI + LPVr	AZT/ABC + 3TC + LPVr	TDF + 3TC/FTC + LPVr
**Year of second-line ART initiation**								
2004 to 2008	684.3	61 (12.5)	8.9 (6.9–11.5)	1	1	1	1	1
2009 to 2010	915.7	165 (22.7)	18.0 (15.5–21.0)	2.0 (1.5–2.7)	3.6 (2.6–4.9)	5.1 (3.4–7.1)	1.2 (0.5–2.8)	0.9 (0.3–2.5)
2011 to 2013	2490.7	127 (7.0)	5.1 (4.3–6.1)	0.6 (0.4–0.8)	2.6 (1.7–4.1)	5.7 (1.0–32.2)	0.7 (0.3–1.6)	1.0 (0.4–2.5)
**Initial second-line regimen**								
AZT + ddI + LPVr	744.1	173 (28.0)	23.2 (20.0–27.0)	1	1			
AZT/ABC + 3TC + LPVr	1528.8	72 (6.5)	4.7 (3.7–5.9)	0.2 (0.2–0.3)	0.2 (0.1–0.4)			
TDF + 3TC/FTC + LPVr	1772.6	107 (8.4)	6.0 (5.0–7.3)	0.3 (0.2–0.3)	0.2 (0.1–0.8)			
ATVr regimen	45.1	1 (3.6)	2.2 (0.3–15.7)	0.1 (0.01–0.7)	0.1 (0.02–3.2)			
**Sex**								
Female	2673.8	237 (11.8)	8.9 (7.8–10.1)	1				
Male	1417.0	116 (11.4)	8.2 (6.8–9.8)	0.9 (0.7–1.1)				
**Age at second-line ART initiation**								
Under 25	262.6	25 (12.8)	9.5 (6.4–14.1)	1				
25 to 29.9	472.7	48 (12.3)	10.2 (7.7–13.5)	1.1 (0.7–1.7)				
30 to 39.9	1853.4	144 (10.6)	7.8 (6.6–9.1)	0.8 (0.5–1.2)				
40 to 49.9	1106.4	102 (12.9)	9.2 (7.6–11.2)	1.0 (0.6–1.5)				
≥50	395.7	34 (11.6)	8.6 (6.1–12.0)	0.9 (0.5–1.5)				
**VL failure on first-line ART**								
Yes	1756.2	145 (11.0)	8.3 (7.0–9.7)	0.9 (0.8–1.2)				
No	2334.5	208 (12.1)	8.9 (7.8–10.2)	1				
**Possible ADR on-first-line ART**								
Yes	1940	183 (13.4)	9.4 (8.2–10.9)	1.2 (0.9–1.5)				
No	2150.7	170 (10.2)	7.9 (6.8–9.2)	1				
**CD4 at second-line ART initiation**								
199.9 cells/µl or less	1295.0	120 (11.9)	9.3 (7.7–11.1)	1	1	1	1	1
200 to 349.9 cells/µl	996.7	103 (14.3)	10.3 (8.5–12.5)	1.1 (0.9–1.4)	1.1 (0.9–1.5)	1.0 (0.7–1.5)	1.2 (0.6–2.5)	1.1 (0.7–1.9)
350 cells/µl or higher	957.6	68 (10.4)	7.1 (5.6–9.0)	0.8 (0.6–1.0)	1.0 (0.7–1.3)	0.9 (0.6–1.4)	1.0 (0.5–2.1)	0.8 (0.4–1.4)
Not measured/missing	841.5	62 (9.6)	7.4 (5.7–9.5)	0.8 (0.6–1.1)	1.5 (1.1–2.2)	1.1 (0.5–2.3)	2.1 (1.1–4.1)	1.4 (0.8–2.6)
**BMI at second-line ART initiation**								
Underweight	316.7	37 (14.7)	11.7 (8.5–16.1)	1				
Normal	1789.2	161 (12.2)	9.0 (7.7–10.5)	0.8 (0.5–1.1)				
Overweight	988.4	82 (11.3)	8.3 (6.7–10.3)	0.7 (0.5–1.0)				
Obese	646.9	47 (9.9)	7.3 (5.5–9.7)	0.6 (0.4–1.0)				
Not measured/missing	349.4	26 (10.2)	7.4 (5.1–10.9)	0.6 (0.4–1.0)				
**Anaemia at second-line ART initiation**								
Yes	620.3	67 (13.1)	10.8 (8.5–13.7)	1.2 (0.9–1.6)	1.3 (1.0–1.7)	1.2 (0.8–1.7)	2.5 (1.4–4.5)	0.9 (0.5–1.6)
No	2701.6	247 (12.9)	9.1 (8.1–10.4)	1	1	1	1	1
Not measured/missing	768.8	39 (6.6)	5.1 (3.7–6.9)	0.6 (0.4–0.8)	0.8 (0.5–1.2)	1.0 (0.3–3.1)	1.5 (0.7–3.2)	0.5 (0.3–0.9)
**AST/ALT ratio at second-line ART initiation**								
<1	353.9	51 (19.4)	14.4 (11.0–19)	1	1	1	1	1
1 to 1.9	1050.9	138 (17.3)	13.1 (11.1–15.5)	0.9 (0.7–1.3)	0.9 (0.6–1.2)	0.8 (0.5–1.1)	1.7 (0.6–5.1)	1.3 (0.6–2.9)
2 or higher	172.9	16 (10.8)	9.3 (5.7–15.1)	0.6 (0.4–1.1)	0.7 (0.4–1.2)	1.0 (0.5–1.9)	0.7 (0.1–6.4)	0.4 (0.1–1.9)
Not measured/missing	2512.9	148 (8.1)	5.9 (5.0–6.9)	0.4 (0.3–0.6)	0.7 (0.5–1.1)	0.7 (0.4–1.2)	1.3 (0.4–4.0)	1.0 (0.5–2.0)
**WHO at second-line ART initiation**								
I or II	2443.0	219 (12.3)	9 (7.9–10.2)	1				
III or IV	722.0	70 (12.8)	9.7 (7.7–12.3)	1.1 (0.8–1.4)				
Not measured/missing	925.7	64 (9.2)	6.9 (5.4–8.8)	0.8 (0.6–1.0)				
**VL failure in follow-up**								
Yes	351.2	29 (14.2)	8.3 (5.7–11.9)	1.0 (0.7–1.4)				
No	3739.5	324 (11.5)	8.7 (7.8–9.7)	1				
**≥3 months late for scheduled visit in follow-up**								
Yes	496.5	23 (7.7)	4.6 (3.1–7.0)	0.5 (0.3–0.8)	0.4 (0.3–0.6)	0.4 (0.2–0.7)	0.5 (0.2–1.5)	0.3 (0.1–0.8)
No	3594.2	330 (12.1)	9.2 (8.2–10.2)	1	1	1	1	1
**Possible ADR in follow up**								
Yes	629.3	88 (21.1)	14.0 (11.3–17.2)	1.8 (1.4–2.3)	1.6 (1.2–2.0)	1.5 (1.1–2.1)	1.9 (1.1–3.4)	1.5 (0.9–2.5)
No	3461.4	265 (10.2)	7.7 (6.8–8.6)	1	1	1	1	1
**Clinic type**								
HIV clinic in hospital complex	2136.5	211 (13.4)	9.9 (8.6–11.3)	1	1	1	1	1
Local CHC	622.9	43 (9.4)	6.9 (5.1–9.3)	0.7 (0.5–1.0)	0.9 (0.6–1.2)	1.1 (0.7–1.9)	0.7 (0.4–1.5)	0.8 (0.5–1.5)
NGO clinic	1331.3	99 (10.0)	7.4 (6.1–9.1)	0.8 (0.6–1.0)	0.9 (0.7–1.1)	0.8 (0.5–1.2)	0.6 (0.3–1.2)	1.1 (0.7–1.7)

Overall, the risk of drug substitution was higher after 2009 as compared to the 2004–2008 cohorts, the hazard ratio for drug substitution was 3.6 (95% CI: 2.6–4.9) for the 2009–2010 cohorts and 2.6 (95% CI: 1.7–4.1) for the 2011–2013 cohorts. This is only true however for patients initiated on AZT + ddI + LPVr for second-line in 2009–2010 (aHR 5.1, 95% CI: 3.4–7.1) and 2011–2013 (aHR 5.7, 95% CI: 1.7–32.2) when ddI was being phased out. Among patients initiated AZT/ABC + 3TC + LPVr or TDF + 3TC/FTC + LPVr for second-line, rates of drug substitutions remained low over time.

Additional predictors of early drug substitutions were CD4 at switch to second-line ART, anaemia, reporting a possible ADR and being late for a scheduled visit during follow-up period. Patients with missing CD4 data around the time of initiating second-line ART were at higher risk of drug substitution compared to those with low CD4 values (<200 cell/µl), particularly those initiated on AZT/ABC + 3TC + LPVr (aHR 2.1, 95% CI: 1.1–4.1). Patients who were anaemic at the time of switch were at higher risk of a drug substitution compared to those with normal haemoglobin levels, particularly those initiated on AZT/ABC + 3TC + LPVr (aHR 2.5, 95% CI: 1.4–4.5).

Patients who experienced an ADR during the observation period were more likely to have been switched following an ADR on first-line ART (OR 1.4, 95% CI: 1.2–1.8). Furthermore, experiencing a possible ADR during follow-up increased the risk of a drug substitution (aHR 1.6, 95% CI: 1.2–2.0). However patients who were late for a scheduled visit had a lower risk of drug substitution compared to those who attended the clinics on time (aHR 0.4, 95% CI: 0.3–0.6).

### Predictors of treatment interruptions

Table 4 presents crude and adjusted hazards ratios (HR & aHR) of treatment interruptions in the first 24 months on second-line ART. As depicted in Figure 2, the risk of treatment interruption was lower among the 2009–2010 (aHR 0.7, 95% CI: 0.5–0.9) and 2011–2013 cohorts (aHR 0.5, 95% CI: 0.3–0.7) compared to the 2004–2008 cohorts. Younger patients (<25 years) were at higher risk of treatment interruptions than older patient (40 to 49 years old) (aHR 0.5, 95% CI: 0.3–0.8). Patients initiated on AZT + ddI + LPVr were at lower risk of treatment interruptions compared to those taking AZT/ABC + 3TC + LPVr (aHR 1.4, 95% CI: 1.0–2.0) or TDF + 3TC/FTC + LPVr (aHR 1.5, 95% CI: 1.0–2.2). Patients for whom AST/ALT ratio was not measured were at lower risk of treatment interruption compared to those with an AST/ALT ratio less than one (aHR 0.6, 95% CI: 0.4–1.0). Having a possible ADR during follow-up increased the risk of early treatment interruption (aHR 1.3, 95% CI: 1.0–1.8). Compared to patients initiated on second-line at a hospital based clinic, those receiving HIV care at a local CHC were at lower risk (aHR 0.6, 95% CI: 0.4–1.0). Additionally having been late for a scheduled visit during follow-up increased the risk of treatment interruptions (aHR 2.8, 95% CI: 2.1–3.7).

**Table 4.** Crude and adjusted predictors of treatment interruption the first two years on second-line ART.

	Crude associations				Adjusted associations
	Person-time	Failures, *n* (%)	Incidence rate/100PY (95% CI)	HR (95% CI)	aHR (95% CI)
**Year of second-line ART initiation**					
2004 to 2008	634.7	80 (16.4)	12.6 (10.1–15.7)	1	1
2009 to 2010	992.2	75 (10.3)	7.6 (6.0–9.5)	0.6 (0.4–0.8)	0.7 (0.5–0.9)
2011 onwards	2487	105 (5.8)	4.2 (3.5–5.1)	0.3 (0.2–0.4)	0.5 (0.3–0.7)
**Initial second-line regimen**					
AZT + ddI + LPVr	813.7	77 (12.5)	9.5 (7.6–11.8)	1	1
AZT/ABC + 3TC + LPVr	1496.7	83 (7.5)	5.5 (4.5–6.9)	0.6 (0.4–0.8)	1.4 (1.0–2.0)
TDF + 3TC/FTC + LPVr	1763.3	96 (7.5)	5.4 (4.5–6.6)	0.6 (0.4–0.8)	1.5 (1.0–2.2)
ATVr regimen	40.3	4 (14.3)	9.9 (3.7–26.5)	1.1 (0.4–2.9)	1.8 (0.6–5.2)
**Sex**					
Female	2691.9	175 (8.7)	6.5 (5.6–7.5)	1	
Male	1422.0	85 (8.3)	6.0 (4.8–7.4)	0.9 (0.7–1.2)	
**Age at second-line ART initiation**					
Under 25	257.5	23 (11.8)	8.9 (5.9–13.4)	1	1
25 to 29.9	471.6	37 (9.5)	7.8 (5.7–10.8)	0.9 (0.5–1.5)	0.7 (0.4–1.2)
30 to 39.9	1848.8	117 (8.6)	6.3 (5.3–7.6)	0.7 (0.5–1.1)	0.6 (0.4–0.9)
40 to 49.9	1134.4	57 (7.2)	5.0 (3.9–6.5)	0.6 (0.4–0.9)	0.5 (0.3–0.8)
≥50	401.7	26 (8.8)	6.5 (4.4–9.5)	0.7 (0.4–1.3)	0.7 (0.4–1.2)
**VL failure on first-line ART**					
Yes	1768.8	109 (8.3)	6.2 (5.1–7.4)	0.9 (0.7–1.2)	
No	2345.1	151 (8.8)	6.4 (5.5–7.6)	1	
**Possible ADR on first-line ART**					
Yes	1956.5	128 (9.4)	6.5 (5.5–7.8)	1.1 (0.9–1.4)	
No	2157.4	132 (7.9)	6.1 (5.2–7.3)	1	
**CD4 at second-line ART initiation**					
199.9 cells/µl or less	1303.0	96 (9.5)	7.4 (6.0–9.0)	1	1
200 to 349.9 cells/µl	1009.4	72 (10.0)	7.1 (5.7–9.0)	1.0 (0.7–1.3)	1.0 (0.7–1.3)
350 cells/µl or higher	943.0	56 (8.6)	5.9 (4.6–7.7)	0.8 (0.6–1.1)	0.8 (0.6–1.1)
Not measured/missing	858.5	36 (5.6)	4.2 (3.0–5.8)	0.6 (0.4–0.8)	1.0 (0.6–1.6)
**BMI at second-line ART initiation**					
Underweight	326.3	23 (9.1)	7.0 (4.7–10.6)	1	
Normal	1803.6	106 (8.0)	5.9 (4.9–7.1)	0.8 (0.5–1.3)	
Overweight	1001.2	56 (7.7)	5.6 (4.3–7.3)	0.8 (0.5–1.3)	
Obese	636.6	55 (11.6)	8.6 (6.6–11.3)	1.2 (0.8–2.0)	
Not measured/missing	346.2	20 (7.9)	5.8 (3.7–9.0)	0.8 (0.5–1.5)	
**Anaemia at second-line ART initiation**					
Yes	628.0	47 (9.2)	7.5 (5.6–10)	1.1 (0.8–1.5)	1.0 (0.7–1.3)
No	2714.0	186 (9.7)	6.9 (5.9–7.9)	1	1
Not measured/missing	771.9	27 (4.6)	3.5 (2.4–5.1)	0.5 (0.3–0.8)	0.7 (0.4–1.6)
**AST/ALT ratio at second-line ART initiation**					
<1	366.1	33 (12.5)	9.0 (6.4–12.7)	1	1
1 to 1.9	1046.9	101 (12.6)	9.6 (7.9–11.7)	1.1 (0.7–1.6)	1.0 (0.7–1.5)
2 or higher	172.1	13 (8.8)	7.6 (4.4–13.0)	0.8 (0.4–1.6)	0.8 (0.4–1.5)
Not measured/missing	2528.8	113 (6.2)	4.5 (3.7–5.4)	0.5 (0.3–0.7)	0.6 (0.4–1.0)
**WHO at second-line ART initiation**					
I or II	2446.9	161 (9.0)	6.6 (5.6–7.7)	1	1
III or IV	731.7	55 (10.0)	7.5 (5.8–9.8)	1.1 (0.8–1.6)	1.1 (0.8–1.6)
Not measured/missing	935.3	44 (6.3)	4.7 (3.5–6.3)	0.7 (0.5–1.0)	0.9 (0.6–1.2)
**VL failure in follow-up**					
Yes	343.6	15 (7.6)	4.4 (2.6–7.2)	0.7 (0.4–1.2)	
No	3770.4	245 (8.7)	6.5 (5.7–7.4)	1	
**≥3 months late for scheduled visit in follow-up**					
Yes	445.3	69 (11.4)	15.5 (12.2–19.6)	3.0 (2.3–3.9)	2.8 (2.1–3.7)
No	3668.6	191 (7.0)	5.2 (4.5–6.0)	1	1
**Possible ADR in follow up**					
Yes	636.3	59 (14.1)	9.3 (7.2–12.0)	1.6 (1.2–2.2)	1.3 (1.0–1.8)
No	3477.6	201 (7.7)	5.8 (5–6.6.0)	1	1
**Clinic type**					
HIV clinic in hospital complex	2161.8	144 (9.1)	6.7 (5.7–7.8)	1	1
Local CHC	639.6	21 (4.6)	3.3 (2.1–5.0)	0.5 (0.3–0.8)	0.6 (0.4–1.0)
NGO run HIV clinic	1312.6	95 (9.6)	7.2 (5.9–8.8)	1.1 (0.8–1.4)	1.3 (1.0–1.8)

## Discussion

The South African HIV treatment programme has evolved substantially since 2004. This is the first paper to examine in detail the impact of guideline changes on rates and predictors of early drug substitutions (in the first 24 months) and treatment interruptions after switching to second-line ART, over the first 11 years of South Africa’s national treatment programme. Among patients initiated on second-line between 2004 and 2013, 11.7% had at least one drug substituted in the first 24 months on second-line, at an overall rate of 8.6 per 100PY. Early drug substitution considerably increased in 2009 and early 2010 among patients initiated on ddI-based second-line regimen and then gradually decreased in for the later second-line cohorts. Treatment interruption on the other hand consistently decreased over time.

The rate of early drug substitution for patients initiated on TDF + 3TC/FTC + LPVr is similar to the reported rate for patients on TDF based first-line regimens (6.3 per 100PY) [23,26]. Drug substitutions among patients on AZT/ABC + 3TC + LPVr are much lower than reported rates among AZT based first-line regimen [23]. This is likely because poor tolerance of AZT in first-line ART would be a contraindication for AZT in second-line. However missing CD4 data and being anaemic at the time of switch were important predictors of drug substitution among patients initiated on AZT/ABC + 3TC + LPVr, indicating exacerbated or unresolved anaemia [16].

Early drug substitutions were mainly driven by ddI substitutions centred around 2010, indicating a rapid phasing out of ddI and transition to newly available drugs as per the 2010 ART guidelines [20,27]. The 2010 guidelines were published and implemented in April 2010, therefore ddI substitutions in the 2010 cohort would have occurred soon after switch, later among patients switched before 2009, hence the longer median time to substitution. Substitutions of TDF, AZT, or ABC occurred within six month of switching to second-line ART suggesting improved access to alternative antiretroviral drugs after 2010.

Drug substitutions were also associated with ADR experiences on second-line ART. In general ADR experiences increase the risk of treatment non-adherence and ART failure, and unresolved non-adherence on first-line ART is an important predictor of VL failure on second-line [12,13,28,29]. We found that patients who experienced an ADR during the observation period were more likely to have been switched following an ADR on first-line ART. Considering the challenges associated with switching patients to third line regimen in South Africa, ADR related VL failure on second-line may prompt drug substitutions. There was however no association between VL failure while on first-line ART or during the observation period with drug substitutions on second-line ART.

The decrease in treatment interruptions over time highlights improvements in continuity of care among second-line ART patients after guideline changes in South Africa. The 2004 to 2008 rate of second-line ART interruptions is substantially higher than the reported rates for the general population on ART in the same period [25], suggesting a higher defaulting rate among second-line ART patients. The proportion lost to follow up among second-line ART patients for the 2004 to 2008 cohort is higher than previously reported rates of losses among first-line patient in the same period [30,31]. However, similar to treatment interruption rates, attrition among second-line ART patients also decreased over time.

As hypothesized, ADR experiences increased the risk of treatment interruptions. While improvements in ART monitoring in South Africa have been noted over the years, ADRs are still under-reported, thus limiting our ability to explain changes in treatment interruptions more accurately [32]. The risk of treatment interruptions was higher among younger patients and those receiving care at NGO clinic compared to hospital-based ART clinics. Being initiated at local CHC was associated with a lower risk, possibly because of the lower burden of second-line ART patients requiring follow-up efforts and the ease of access to CHC facilitating patient retention. Younger age and being homeless have previously been associated with treatment interruptions [24]. While other studies in South Africa have reported being male to be a predictor of treatment interruptions [25], there was no gender difference in this analysis

The interpretation of these results is limited to the context from which participants were drawn. The data for this analysis were drawn from a sample of public/NGO health facilities in Johannesburg and patients included may not be representative of patients across South Africa. Furthermore treatment interruption (as a proxy for continuity of care) may have been underestimated as only treatment breaks involving regimen changes was considered. The analysis does not account for treatment interruption within ART regimen. The lack of complete information on the clinical reasons for the drug substitutions or treatment interruptions further limit our ability to accurately examine drug toxicity related predictors of early drug substitutions among patients initiated on second-line regimen.

## Conclusions

Our results show a relatively rapid transition between 2004 and 2010 ART guidelines, highlighting the health system’s responsiveness to changes in HIV treatment policies and improved continuity of care among second-line ART patients. Drug toxicity reporting and monitoring systems, particularly among second-line ART patients, need improvements to inform timely regimen changes and ensure that patients remain in care. However reasons for drug substitutions should still be closely monitored to ensure that patients do not run out of treatment options in the future.
